# A Bifunctional Nanostructured RuPt/C Electrocatalyst for Energy Storage Based on the Chlor-Alkali Process

**DOI:** 10.3390/nano15070506

**Published:** 2025-03-27

**Authors:** Nuria Romero, Mahmoud M. Gomaa, Jérôme Esvan, Manuel A. Rodrigo, Karine Philippot, Justo Lobato

**Affiliations:** 1 LCC (Laboratoire de Chimie de Coordination), 205 Route de Narbonne, BP 44099, 31077 Toulouse, Cedex 4, France; karine.philippot@lcc-toulouse.fr; 2 Université de Toulouse, UPS, INPT, 118 Route de Narbonne, 31400 Toulouse, France; 3 Physics Department, Faculty of Science, Minia University, Minia P.O. Box 61519, Egypt; mahmoud.mohammed@uclm.es; 4 Chemical Engineering Department, Faculty of Chemical Sciences and Technologies, Universidad Castilla-La Mancha, 13004 Ciudad Real, Spain; manuel.rodrigo@uclm.es; 5 CIRIMAT, Université de Toulouse, CNRS-INPT-UPS, 4 Allée Emile Monso, BP 44362, 31030 Toulouse, France; jerome.esvan@toulouse-inp.fr

**Keywords:** bimetallic RuPt, nanoparticles, chlor-alkali, clean energy, unitized–reversible electrochemical cell

## Abstract

This study focuses on the design of a novel electrode for an energy storage system utilizing EDEN (electrochemical-based decarbonizing energy) technology. This technology implies a chlor-alkali electrochemical cell with dual functionality: first, the electrolysis of water and NaCl to produce hydrogen (H_2_) and chlorine (Cl_2_), and subsequently, the utilization of these products in an H_2_/Cl_2_ fuel cell to generate electricity. Bimetallic RuPt nanoparticles have been synthesized on Vulcan carbon (C-V) from organometallic precursors to be used as electrocatalysts. Characterization includes transmission electron microscopy (TEM), high-angle annular dark-field scanning transmission electron microscopy (HAADF-STEM), energy-dispersive X-ray spectroscopy (EDX), X-ray photoelectron spectroscopy (XPS), and powder X-ray diffraction (XRD). The RuPt/C-V-based electrode demonstrated notable performance in the target reversible electrochemical cell, acting as the anode for electrolysis and as the cathode in fuel-cell mode. Testing in a 3D-printed electrochemical cell revealed high efficiency, with a coulombic efficiency exceeding 96% for hydrogen production, yielding 11.75 mg·Wh^−1^ and achieving a power output of approximately 4.5 mW·cm^−2^ in H_2_/Cl_2_ fuel-cell operation.

## 1. Introduction

Given climate change and its consequences on the planet and humanity, there is a general interest in the use and development of renewable energy technologies [1]. Since most renewable energies are intermittent, one possible solution is to store energy in the chemical bonds of molecules, the so-called solar fuels [2]. Among all, hydrogen is one of the most promising energy vectors [3,4] since it can be obtained through water electrolysis activated via green energy, has a high combustion enthalpy of −285.8 kJ/mol, and releases water as the only product of its combustion. The production of green hydrogen without CO_2_ emission might be the ideal way to replace fossil fuels. It can be used as raw material for chemical transformations like hydrogenation reactions or fertilizer production, and it can also be converted back into electricity [5].

Water splitting (2H_2_O → 2H_2_ + O_2_) is nowadays carried out to produce hydrogen for its usage in fuel cells. Proton-exchange-membrane (PEM) fuel cells, working at a low temperature compared to solid-oxide fuel cells (SOFCs), are the most commonly used technology in commercial devices [6]. There are two main challenges to solve in order to make advances in energy storage through power to X.

The first issue is to overcome the kinetic and thermodynamic barriers of the electrochemical water-splitting process (E^0^_cell_ = −1.23 V), often with high overpotential values and a consequent energy loss. In order to develop a trustable technology, the use of efficient and durable catalysts is a requisite both in the cathode (where hydrogen is produced) and in the anode. Platinum on carbon is the catalyst of choice for commercial devices. Using nanoscale catalysts in the form of metal nanoparticles presents several advantages, among which the high surface/volume ratio is remarkable, as it allows for a reduction in the amount of material used and, therefore, the catalyst prize [7,8,9,10,11,12,13,14,15]. The optimization of nanocatalysts includes the introduction of doping elements on carbon [16,17,18,19,20,21] or the use of bimetallic nanoparticles [22,23,24,25,26], among others. The second issue is to develop solutions for hydrogen storage. Different research is ongoing in this field, exploring the storage of hydrogen in liquid and solid forms [27] or through redox-flow batteries, which allow the storage of energy in chemicals [28,29,30].

Recently, the use of unitized–reversible Cl_2_/H_2_ cells has been explored to balance energy production and demand by integrating chlor-alkali technology with PEM fuel cells in a reversible mode [24,31]. This advanced energy storage system using EDEN (electrochemical-based decarbonizing energy) technology not only represents a significant step forward in enhancing versatility and efficiency by combining energy production and storage within a single device but also has potential applications in CO_2_ fixation and sodium hydroxide production. One notable advantage of this technology is the ability to use saline water instead of purified water. The system operates in two modes: electrolysis mode (to store energy) and fuel-cell mode (to generate electricity).

In electrolysis mode, electrical energy is applied to split saline water, producing H_2_, Cl_2_, and sodium hydroxide (NaOH).

At the anode: 2NaCl → Cl_2_ + 2e^–^ (1)

at the cathode: 2H_2_O + 2e^−^ ↔ H_2_ + 2OH^−^ (2)

Overall reaction: 2NaCl(aq) + 2H_2_O(l) → 2NaOH(aq) + Cl_2_(g) + H_2_(g) (3)

In fuel-cell mode, stored chlorine and hydrogen react to generate electricity according to the following reaction:

H_2(g)_ + Cl_2(g)_ ↔ 2HCl _(aq)_       (E_0_ = 1.36 V) (4)

In this work, the synthesis and characterization of a RuPt bimetallic nanocatalyst supported onto Vulcan carbon was carried out for the first time through the organometallic approach [32], which allows precise control and tunability for the surface, size, and composition of the nanoparticles obtained. Moreover, the nanomaterial synthesized was used in the cathode of a unitized–reversible H_2_/Cl_2_ electrochemical cell for both the electrolysis and fuel-cell modes.

## 2. Materials and Methods

All synthesis and reagent-handling procedures were accomplished using standard Schlenk and Fisher–Porter tube techniques or a glovebox workstation. High-purity Ar and H_2_ gases were purchased from Air Liquide. The solvents (THF and pentane) were purified before use via filtration with adequate columns in a purification apparatus (MBraun). The water content in solvents was determined with a Karl Fisher Coulometer (Metrohm) (target value < 5 ppm) and then degassed using the freeze–pump–thaw method before use. Precursors (1,5-cyclooctadiene)(1,3,5-cyclooctatriene) Ruthenium (0) (abbreviated as [Ru(COD)(COT)]) and tris(norbornene) Platinum (0) (abbreviated as [Pt(NBE)_3_]) were purchased from Nanomeps. Vulcan carbon xc-72-r was obtained from Fuel Cell Store and dried under vacuum before use. Nafion ionomer solution was purchased from Sigma Aldrich.

### 2.1. Synthetic Procedure

RuPt/C-V was prepared following the organometallic approach. Briefly, a Fisher–Porter containing Vulcan carbon (75 mg) was charged with the two organometallic precursors [Pt(NBE)_3_] (24.9 mg, 0.05 mmol) and [Ru(COD)(COT)] (47.3 mg, 0.15 mmol) and dissolved in 50 mL of THF. Then, this suspension was exposed to 3 bars of H_2_ under dynamic flow for 10 min and under static flow overnight under vigorous stirring. The black suspension obtained was decanted and washed with 75 mL of pentane 3 times. Finally, the resulting powder was dried under reduced pressure to yield 102 mg of dried RuPt nanomaterial. Carbon-covered copper grids were prepared from the colloidal suspension at every stage of the procedure (right after synthesis and from a suspension prepared with the purified and dried nanomaterial). TEM, HRTEM, and STEM analysis were performed on this grid. The mean size of the nanoparticles was 1.8 ± 0.3 nm, and 18.4% wt Pt and 26.5% wt. Ru was determined via ICP-OES applied to the dry powder. The powder was subjected to slow air diffusion before use in catalysis to create a passivation shell on the nanoparticles and avoid the spontaneous pyrolysis of the material.

### 2.2. Electrochemical Cell Design

**Figure 1:**
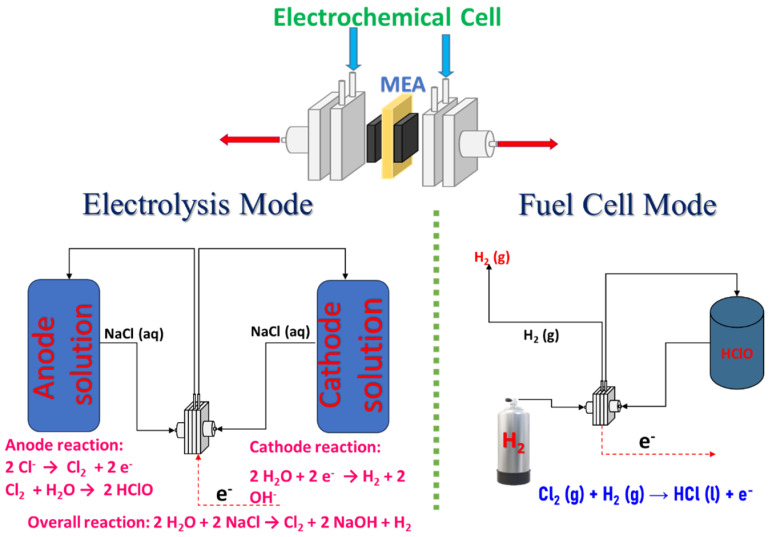
(**top**) Design of the electrochemical cell and (**bottom**) scheme of the experimental setup used in electrolysis mode and fuel-cell mode.

The electrochemical cell was designed and fabricated using 3D-printing technology. It includes two compartments, one for the anode solution and the other for the cathode solution, as well as a central slot to hold the membrane–electrode assembly (MEA). The anode–membrane–cathode assembly was prepared in a “zero-gap” configuration, ensuring direct contact between the three components without any separation. The cell has two bifunctional electrodes, each alternating roles based on the operating mode. During electrolysis, one electrode serves as the anode for chloride oxidation, while in fuel-cell mode, this electrode acts as the cathode for chlorine reduction. The electrochemical cell used is shown in Figure 1.

The preparation of the first electrode (acting as the anode during the electrolysis and as the cathode in fuel-cell mode) supported by a titanium (Ti) felt layer required different steps. First, the Ti felt sheet underwent chemical cleaning following established procedures [33]. This process involved chemical treatments using 20% (*w*/*w*) hydrochloric acid (HCl) and 10% (*w*/*w*) oxalic acid, each for 15 min at 80 °C. This cleaning process removed oxidation layers and surface impurities, leaving the Ti substrate clean and free of contamination. Then, the RuPt/C-V catalyst powder was air-sprayed onto the Ti surface. The catalyst ink used for this step was prepared using isopropanol as the solvent and a 5 wt.% Nafion ionomer solution to ensure uniform dispersion and optimal catalytic performance. The metal loading was 0.5 mg·cm^−2^.

The second electrode, functioning as the cathode during electrolysis and as the anode in fuel-cell mode, was a carbon cloth on top of which a catalyst layer was sprayed. A Pt/C commercial catalyst (Fuel Cell Store) containing 40% platinum supported on carbon black was used as catalytic material. The catalyst was mixed with a 5 wt.% Nafion ionomer solution dissolved in isopropanol to produce a homogeneous catalyst ink. This ink was applied onto a carbon cloth substrate through air-spraying, ensuring a controlled and uniform platinum loading of 0.2 mg Pt·cm^−2^. Additional details on this preparation procedure are available in a previously published study [34].

### 2.3. Characterization

#### 2.3.1. Electron Microscopy Analysis

Transmission electron microscopy (TEM), high-resolution transmission electron microscopy (HR-TEM), high-angle annular dark-field coupled with scanning transmission electron microscopy (HAADF-STEM), and energy-dispersive X-ray scattering (EDX) analyses were performed at the Centre de Microcaractérisation Raimond Castaing, CNRS-UAR 3623, Toulouse, either on a JEOL JEM 1400 operating at 120 kV with a point resolution of 2.0 Å or on a JEOL JEM-ARM200F Cold FEG operating at 200 kV with a point resolution of 1.9 Å, respectively. Samples were prepared by dropcasting a colloidal suspension onto a carbon-covered copper grid. The size distributions and mean sizes of the NPs were determined by a measurement of at least 200 individual nanoparticles on a given grid using the Image J software. The results are given as mean size ± standard deviation.

#### 2.3.2. ICP-OES

Inductively coupled Plasma with optical emission spectroscopy (ICP-OES) was performed at the “Laboratoire de Chimie de Coordination, Toulouse” using a Thermo Scientific ICAP 6300 instrument after the digestion of the metals for at least one night in a 3:1 mixture of HCl:HNO_3_.

#### 2.3.3. XPS

The photoelectron emission spectra were recorded using a monochromatized Al Kalpha (hν = 1486.6 eV) source on a ThermoScientific K-Alpha system at the CIRIMAT-Toulouse laboratory. The X-ray spot size was about 400 µm. The pass energy was fixed at 30 eV with a step of 0.1 eV for core levels and 100 eV for surveys with a step of 1 eV. The spectrometer energy calibration was conducted using the Au 4f7/2 (83.9 ± 0.1 eV) and Cu 2p3/2 (932.8 ± 0.1 eV) photoelectron lines. XPS spectra were recorded in direct mode N (Ec), and the background signal was removed using the Shirley method. Data processing was performed using Avantage software. The flood gun was used to neutralize charge effects on the top surface. Calibration spectra were applied with C1s’ binding energy at 284.5 (±0.1 eV).

#### 2.3.4. XRD

XRD analyses were performed using a Philips PW-1700 diffractometer equipped with CuKα radiation. The 2θ angular regions from 0 to 100° were explored at a scan rate of 0.1°s^−1^. The Scherrer equation was used to calculate the platinum crystallite size (Equation (5)):

(5) $$L_{C}=\frac{0.89\;\lambda \;}{\mathrm{\beta cos}\left(\theta \right)}$$

where *L_C_* is the crystallite size in nm, λ corresponds to the Kα radiation of copper (λ = 0.15418 nm), β is a parameter related to the full width at half the maximum intensity of the peak, and θ is the angle corresponding to I_max_(rad).

References for Ru, RuO_2_, and Pt were taken from the ICSD database under the PDF numbers 01-070-0274, 01-071-2273, and 01-087-0636, respectively.

## 3. Results and Discussion

### 3.1. Synthesis and Characterization of the Materials

**Scheme 1:**
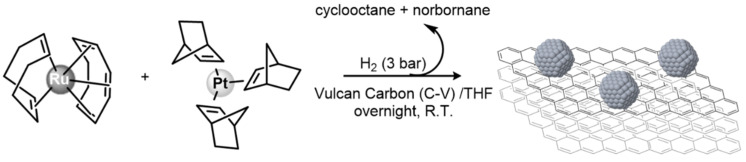
Synthesis of RuPt/C-V via hydrogen treatment of [Pt(NBE)_3_] and [Ru(COD)(COT)] organometallic complexes in the presence of Vulcan carbon.

**Figure 2:**
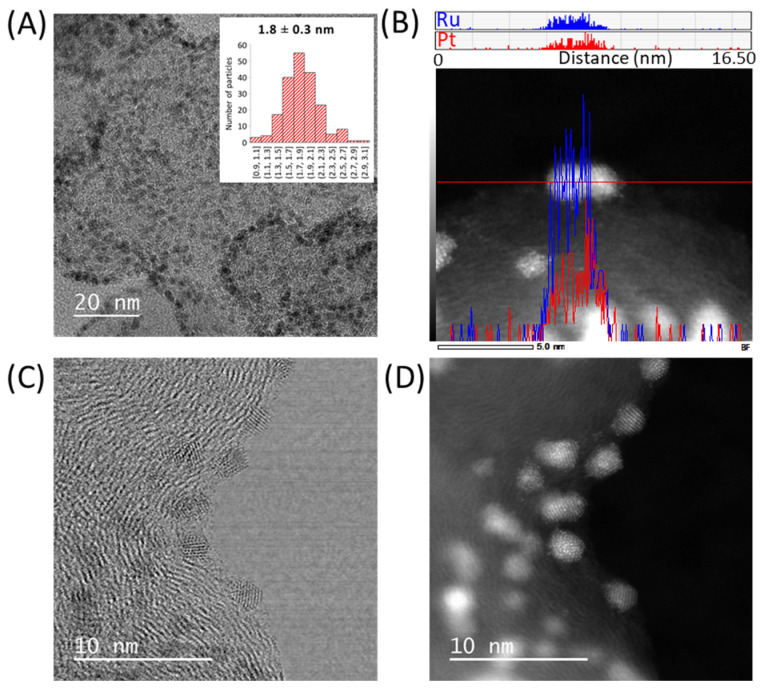
Electron microscopy characterization of RuPt/C-V nanomaterial: (**A**) TEM with mean-size histogram analysis, (**B**) line EDX analysis (Ru in blue; Pt in red), (**C**) HR-TEM, and (**D**) HAADF-STEM.

Bimetallic RuPt nanoparticles supported on Vulcan carbon, **RuPt/C-V**, were synthesized following the organometallic approach [32]. Two organometallic precursors, namely [Pt(NBE)_3_] and [Ru(COD)(COT)], were introduced in a Fischer porter reactor containing Vulcan carbon under an inert atmosphere (Scheme 1). Dry and degassed THF was added as the solvent of the reaction. The reaction mixture then was exposed overnight under 3 bars of pure H_2_ at room temperature. This treatment led to the decomposition of the organometallic precursors through the hydrogenation of the native ligands into alkanes (cyclooctane and norbornane), which are inert and soluble, and then the release of naked metal atoms, which are unstable and tend to nucleate fast on the surface of Vulcan carbon. When the reaction was finished, excess hydrogen was removed from the Fischer porter bottle and replaced with Ar. A TEM grid was prepared by dropcasting one drop of the colloidal suspension onto a carbon-covered copper grid. TEM analysis confirmed the formation of small nanoparticles of 1.8 ± 0.3 nm (Figure 2A). Pentane was added to the THF colloidal suspension in order to precipitate the RuPt nanomaterial, which was then washed several times under inert conditions and dried under a vacuum, leading to a fine black powder. Given that the only subproducts formed during the reaction, cyclooctane and norbornane, are soluble in pentane and volatiles, they were eliminated during the washing and vacuum-drying of the powder [32]. Therefore, it is reasonable to conclude that the RuPt NPs are free of ligands, with the Vulcan carbon support partially covering their surface. The slow passivation of RuPt/C-V powder was performed through exposure under diluted air over 3 weeks at room temperature to avoid any violent reaction and the burning of the nanoparticles (with a consequent loss of its nanostructure), a protocol already shown to be efficient in a previous work involving Ru NPs [35]. The TEM analysis performed from a prepared suspension of the passivated RuPt/C-V nanomaterial showed no significant changes to the size and shape of the nanoparticles (see Figure S2 in the Supporting Information).

The choice of metal precursors is known to be critical for the formation of a mixture of monometallic nanoparticles, core-shell bimetallic nanoparticles, or alloys [36,37]. Here, the hydrogenation rates of [Pt(NBE)_3_] and [Ru(COD)(COT)] complexes follow similar kinetic profiles (some minutes are enough to start forming nuclei and clusters) [38], thus favoring the co-nucleation of naked Ru and Pt atoms into bimetallic alloys, rather than core-shell nanoparticles or a mixture of monometallic particles. An EDX analysis yielded clear evidence of the formation of bimetallic RuPt NPs. Although atomic percentages calculated via EDX are not precise, the results always indicated the presence of both metals in the analysis of several individual nanoparticles (see Figure S3 in the Supporting Information). EDX mapping (Figure S4) also showed the presence of both metals on the overall carbonaceous support. Furthermore, line EDX analysis (Figure 2B) showed that both metals are present in the same nanoparticle. Although the technique is limited by the small size of the nanoparticles, the results of the line EDX strongly suggest that the structure is alloyed, rather than core-shell, which is in agreement with the use of metal precursors that have similar hydrogenation rates. The same conclusion was obtained when Cerezo-Navarrete et al. [39] prepared alloy-type nanoparticles on nitrogen-doped reduced-graphene oxide (NH_2_-rGO), using the same precursors in different ratios. Interestingly, the RuPt NPs obtained using NH_2_-rGO had mean sizes ranging from 1.5 to 2.5 nm, similar to the nanoparticle prepared in this work (1.8 ± 0.3 nm). This fact highlights that the nature of the carbon support is not very relevant to the nucleation and growth of RuPt NPs under the applied conditions. The electron-diffraction patterns of isolated nanoparticles were not conclusive, as there is not any known diffraction pattern for RuPt bimetallic nanoparticles in the database.

ICP-OES analysis allowed a determination of the metal composition of the RuPt/C-V nanomaterial. The results indicated a 26.3% wt. of Ru and an 18.4% wt. of Pt, which corresponds to a ratio of 2.8:1 for Ru/Pt, which is close to the desired ratio of 3:1. This specific ratio was selected, as our previous study showed that a Ru/Pt ratio between 3 and 4 enhances the efficiency of product formation in reversible chlor-alkali cells [40].

**Figure 3:**
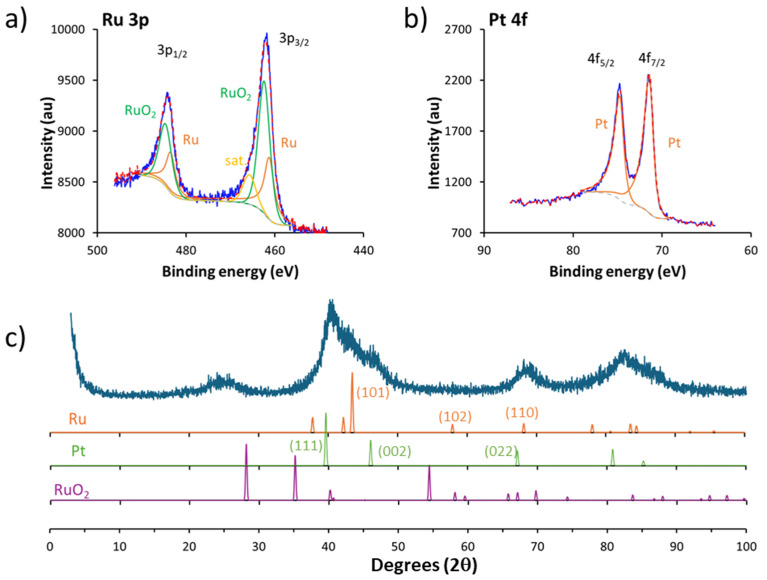
XPS and p-XRD characterization of RuPt/C-V nanomaterial: (**a**) Ru 3p XPS, (**b**) Pt 4f XPS, and (**c**) p-XRD diffractograms.

The oxidation states of Ru and Pt in RuPt/C-V as synthesized and exposed to ambient conditions were determined via X-ray photoelectron spectroscopy (XPS) (Figure S5). The Ru 3p and Pt 4f regions are depicted in Figure 3a and Figure 3b, respectively. In the Ru 3p_3/2_ signal deconvolution, it is possible to identify Ru(0) and RuO_2_ species at 461.2 and 462.4 eV, respectively [41], which indicates that the sample was partially oxidized under air (with a RuO_2_/Ru ratio of ca. 1.7). This behavior is commonly observed for Ru NPs, but generally, they are reduced back to Ru(0) under HER turnover conditions [35]. On the other hand, the Pt 4f7/2 signal at 71.4 eV corresponds to metallic Pt, as expected [42], indicating that it is stable under air conditions. It is important to highlight that, with less than 2 nm-size nanoparticles, this analysis represents not only the surface but also the entire nanoparticle oxidation state.

The powder XRD pattern of the RuPt/C-V nanomaterial is shown in Figure 3c. The diffractogram shows wide signals. This is very common when the nanoparticles have a very small size, as most of the atoms are found at the surface. Additionally, the presence of Vulcan carbon could also be detrimental to registering a high-quality diffractogram. Nevertheless, some features might be identified for metallic Ru and metallic Pt, particularly the addition of the (111) peak of Pt and (101) peak of Ru at 40°. Although clearly detected via XPS, RuO_2_ features were not observed, indicating that it is likely amorphous, which is consistent with the fact that, upon the passivation with air, RuO_2_ is formed at the surface of the nanoparticles.

The Pt lattice parameter was calculated from the Pt (200) diffraction peak position in the XRD pattern, and the values were about 3.923 Å. And the Ru atomic fractions in the alloy were determined according to Vegard’s law [43,44]:

(6) $$X_{Ru}=\frac{0.39155-\alpha_{fcc}}{K}$$

where *X*_Ru_ is the atomic fraction of Ru, 0.39155 is the lattice parameter for pure platinum supported on nanofibres, α_fcc_ is the lattice parameter value, and *K* = 0.0124 nm is an empirical constant. From this calculation, we estimated that the Ru atomic fraction in the alloy was approximately 3.25%. The lattice parameter of pure Pt is well established as 3.923 Å, while the calculated value for the Pt-based electrocatalyst in this work is slightly reduced to 3.919 Å (Δα = 0.004 Å). This contraction likely arises from compressive strain induced via interactions between Pt nanoparticles and the Vulcan carbon support or adjacent RuO_2_ phases, rather than alloying with Ru. Although Ru primarily exists as a separate RuO_2_ phase, as indicated by the absence of significant Pt peak shifts in XRD, its proximity to Pt may modulate the electronic structure of Pt through interfacial electron transfer. Such interactions could lower the Pt d-band center, weakening the adsorption energies of reaction intermediates and thereby enhancing chlorine reduction reaction (CRR) activity.

### 3.2. Electrochemical Performance

#### 3.2.1. Electrolysis Mode

**Figure 4:**
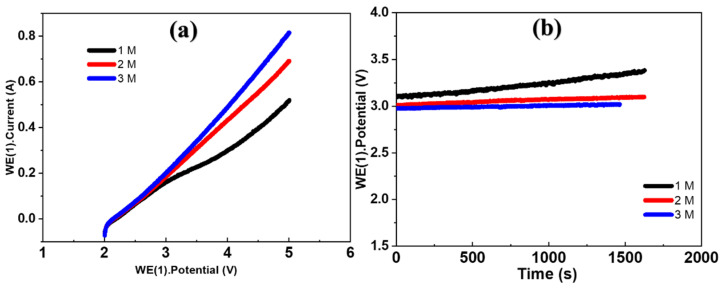
(**a**) Polarization curves of electrolysis mode with RuPt/C-V as the anode and Pt/C (0.2 mg·cm^−2^) as the cathode with Nafion 117 membrane for NaCl brine concentrations of 1, 2, and 3 M; (**b**) voltage at 50 mA·cm^−2^ across various NaCl brine concentrations (1, 2, and 3 M) at 20 °C.

**Figure 5:**
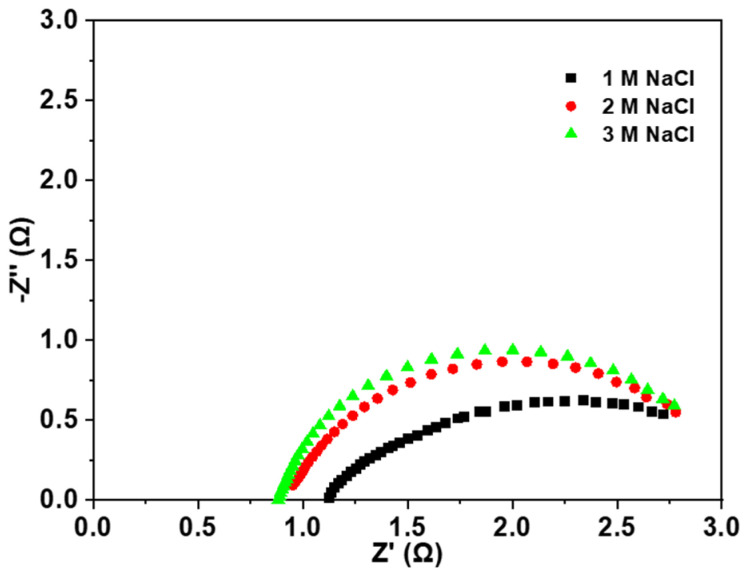
Nyquist plot in electrolysis mode with RuPt/C-V as the anode and Pt/C (0.2 mg·cm^−2^) as the cathode with the Nafion 117 membrane at various NaCl brine concentrations (1, 2, and 3 M) at 20 °C.

The performance of the electrochemical cell in electrolysis mode is illustrated in Figure 4a,b. Figure 4a presents the polarization curves at varying NaCl concentrations. A higher current density was observed at 3 M NaCl for the same applied voltage compared to lower concentrations. This indicates reduced energy consumption under these conditions, highlighting enhanced system efficiency. Furthermore, Figure 4b shows the voltage values at a fixed current density of 50 mA·cm^−2^, clearly revealing that the voltage at 3 M NaCl is lower than that observed at the two lower NaCl concentrations. This reduction in voltage can be attributed to the decrease in resistance with an increasing brine concentration, which was confirmed through in situ electrochemical impedance spectroscopy (EIS) measurements (Figure 5). The data show that the ohmic resistance at 1 M NaCl is 2.1 Ω, and it decreases to 0.8 Ω at 3 M NaCl. This reduction is primarily due to the increased density of mobile charge carriers in the electrolyte, which increases the ionic conductivity [45]. In addition, the higher ionic concentration reduces the impedance of the electrochemical double layer by increasing the availability of ions near the electrode surface [46], allowing for a more efficient transfer of charges. Additionally, at higher NaCl concentrations, the reduction in viscosity improves ion mobility [47], accelerates diffusion, and reduces the accumulation of gas bubbles, issues that are commonly encountered in systems with lower-ionic-strength electrolytes.

In the chlor-alkali process, the generated Cl_2_ and H_2_ gases present practical challenges, particularly the formation of gas bubbles that are trapped between the electrode surface and the membrane–electrode interface. This issue is especially pronounced in zero-gap configurations, where the distance between the membrane and the electrodes is minimal. One key parameter affecting the cell performance is the rate at which gas bubbles are removed. Bubbles that are formed on the electrode surface adhere and grow until they detach. While attached, these bubbles partially block the electrode surface, increasing ohmic resistance and reducing the area available for current and mass transport. Therefore, efficient gas detachment from the electrodes must be ensured.

#### 3.2.2. Hydrogen Production

**Figure 6:**
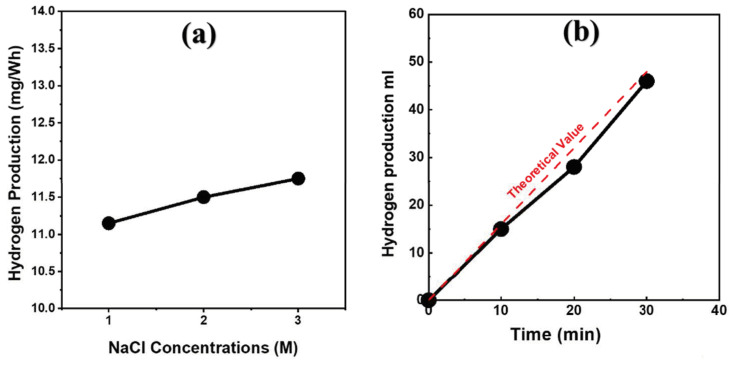
(**a**) H_2_ production in mg·Wh^−1^ using different concentrations of brine solutions at 20 °C; (**b**) H_2_ production (mL) as the function of operating time at electrolysis mode with RuPt/C-V as the anode and Pt/C (0.2 mg·cm^−2^) as the cathode with Nafion 117 membrane at 3 M NaCl brine solutions.

To optimize the electrolyte conditions for maximizing H_2_ production in electrolysis mode, the production of H_2_ catalyzed via RuPt/C-V was measured at different concentrations of brine solutions. As shown in Figure 6a, H_2_ production, expressed as mg·Wh^−1^, increases with increasing NaCl concentrations from 11.1 mg·Wh^−1^ at 1 M of NaCl to 11.8 mg·Wh^−1^at 3 M of NaCl. This behavior is attributed to the direct influence of brine concentration on key parameters, including ionic conductivity, ohmic resistance, energy efficiency, and gas evolution dynamics. As previously discussed, higher brine solution concentrations reduce resistance, facilitating faster charge and ion transfer. This enhancement accelerates the chemical reactions occurring at both the anode and cathode, improving the overall efficiency of the system. For comparison, the Best Available Technologies Reference Document (BREF) from the European Union, which evaluates industrial chlor-alkali cells for H_2_ production, reports production rates of up to 10.63 mg H_2_ Wh^−1^ under optimal conditions, thus showing the good performance of the RuPt/C-V nanocatalyst [48,49]. However, it is important to mention that industrial systems often operate at significantly higher temperatures, approaching the boiling point of brine solution, which further enhances reaction kinetics and performance.

The system performance was further evaluated at a fixed current density of 50 mA·cm^−2^ using 3 M NaCl (Figure 6b). The H_2_ production is increased linearly over time and aligns closely with the theoretical values, achieving a coulombic efficiency of over 96%. This high efficiency demonstrates the high performance of the designed electrochemical cell in electrolysis mode, highlighting its ability to operate with minimal energy losses and optimal hydrogen generation.

#### 3.2.3. Fuel-Cell Mode

The designed electrochemical cell was switched to fuel-cell mode with the H_2_/Cl_2_ system. The hydrogen source was a compressed tank, while chlorine was supplied in the form of hypo-chlorous acid (HClO, pH ~2). Under these operating conditions, the fuel cell acted as a power source by converting chemical energy from H_2_ and HClO into electrical energy.

**Figure 7:**
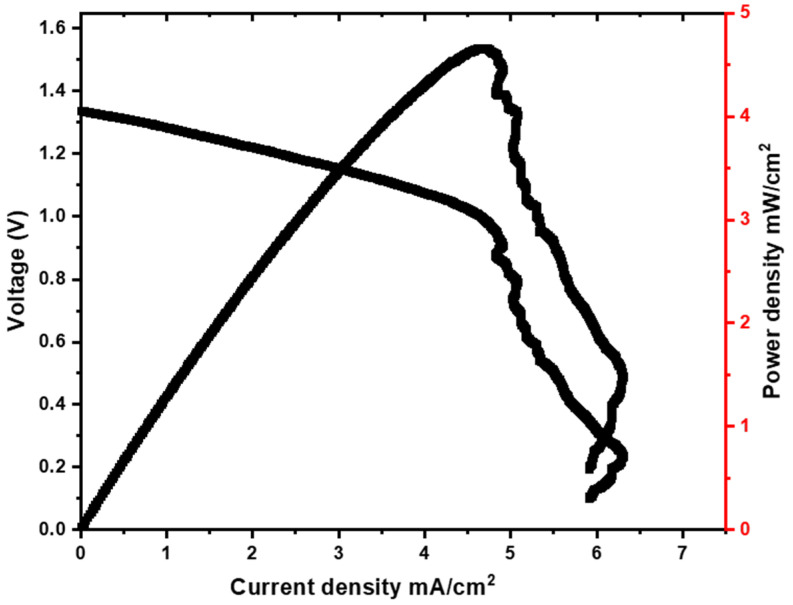
Polarization curves and the obtained power density in H_2_/Cl_2_ fuel-cell mode using the Nafion 117 membrane with RuPt/C-V (0.5 mg·cm^−2^) as the cathode and Pt/C (0.2 mg·cm^−2^) as the anode. The H_2_ rate was 50 mL·min^−1^, and the HClO feeding rate was 30 mL·min^−1^.

Figure 7 shows the performance of the H_2_/Cl_2_ fuel cell using the prepared electrodes at room temperature. The polarization curve has the same characteristic behavior as the conventional H_2_/O_2_ fuel cells, with different ranges corresponding to activation losses, ohmic resistances, and mass transport limitations [50]. The main difference between the H_2_/O_2_ fuel cells and the system of study is the activation-loss regions. In the H_2_/Cl_2_ fuel cell, the activation losses were significantly lower than those typically found in H_2_/O_2_ ones. This improvement can be attributed to the high activity of the chlorine-reduction reaction that takes place at the cathode of the system of study. This reaction is catalyzed by ruthenium oxide (RuO_2_), which is known to facilitate the chlorine-reduction process with an exchange current density of approximately 1 × 10^−2^ mA·cm^−2^ [51]. In contrast, in the H_2_/O_2_ fuel cells, the oxygen-reduction reaction (ORR) on platinum catalysts generally works with a much lower exchange current density, about 1 × 10^−6^ mA·cm^−2^ [52,53]. This higher catalytic activity contributes to faster reaction kinetics, enhancing the overall fuel-cell efficiency and reducing energy losses associated with slow cathode reactions. RuO_2_ plays a significant role in enhancing electrode stability in electrochemical systems. Its high corrosion resistance contributes to electrode durability and long-term performance. For instance, different studies have shown that RuO_2_ exhibits high stability during oxygen and chlorine evolution reactions [53,54], which are critical in various electrochemical processes. The power density achieved in this study was approximately 4.8 mW·cm^−2^, representing a high improvement compared to our previous work [23], where a power density of 1.9 mW·cm^−2^ was obtained using a (RuO_2_)_70_/(Pt)_30_ electrode at the cathode in fuel-cell mode, prepared via the Piccini method. Despite these promising results, several parameters still need to be optimized to maximize the efficiency of the system in both electrolysis and fuel-cell modes. Further research is needed to optimize components such as membrane materials, cell design, and operating parameters to enhance system performance and long-term viability.

## 4. Conclusions

The successful synthesis and thorough characterization of a novel nanomaterial made of RuPt nanoparticles dispersed on carbon Vulcan, prepared through the organometallic approach, were achieved in this work. Nanoparticles of a small size (ca. 1.8 nm) with a clean surface and a narrow size distribution have a RuPt alloy-type structure with a thin layer of RuO_2_ at their surface, formed upon slow air exposure. The electrochemical performance of this nanomaterial was first evaluated for the hydrogen evolution reaction, showing promising results. Then, the nanomaterial was integrated into the cathode of a reversible H_2_/Cl_2_ electrochemical 3D-printed fuel cell. A power density of ca. 4.8 mW·cm^−2^ was achieved, representing a high improvement compared to previously reported catalysts. Thus, the double functionality of this RuPt/C-V nanomaterial for energy storage has been demonstrated. The perspectives of this work included exploring the optimization of the metal ratio and metal loading on carbon and the optimization of the operating parameters of the electrochemical setup when aiming for long-live effective devices.

## Data Availability

The raw data supporting the conclusions of this article will be made available by the authors upon request.

## Supplementary Materials

The following supporting information can be downloaded at https://www.mdpi.com/article/10.3390/nano15070506/s1: Figure S1. TEM (**a**,**b**), HRTEM (**c**), and STEM-HAADF (**d**) analysis of RuPt/C-V nanomaterial at different magnifications; Figure S2. TEM analysis of the RuPt/C-V nanomaterial after passivation (diluted air, room temperature, 3 weeks); Figure S3. EDX analysis of individual RuPt/C-V nanoparticles depicted on a STEM-HAADF image; Figure S4. EDX mapping of RuPt/C-V nanomaterial; Figure S5. XPS survey of RuPt/C-V nanomaterial.
