# Supplementary material for: A Bifunctional Nanostructured RuPt/C Electrocatalyst for Energy Storage Based on the Chlor-Alkali Process
Source: Nanomaterials (Basel). 2025 Mar 27;15(7):506. doi: 10.3390/nano15070506 (PMC11990831; doi:10.3390/nano15070506)
Supplement: Supplementary file 1 [file nanomaterials-15-00506-s001.zip › nanomaterials-3531207-supplementary.pdf]

Supporting Information

A Bifunctional Nanostructured RuPt/C Electrocatalyst for Energy Storage Based on the Chlor-Alkali Process

Nuria Romero [1,2,*,†], Mahmoud M. Gomaa [3,4,†], Jérôme Esvan [5], Manuel A. Rodrigo [4], Karine Philippot [1,2] and Justo Lobato [3,*]

[1] LCC (Laboratoire de Chimie de Coordination), 205 Route de Narbonne, BP 44099, 31077 Toulouse, Cedex 4, France
[2] Université de Toulouse, UPS, INPT, 118 Route de Narbonne, 31400 Toulouse, France
[3] Physics Department, Faculty of Science, Minia University, Minia P.O. Box 61519, Egypt
[4] Chemical Engineering Department, Faculty of Chemical Sciences and Technologies, Universidad Castilla-La Mancha, 13004 Ciudad Real, Spain
[5] CIRIMAT, Université de Toulouse, CNRS-INPT-UPS, 4 Allée Emile Monso, BP 44362, 31030 Toulouse, France
* Correspondence: nuria.romero@lcc-toulouse.fr (N.R.); justo.lobato@uclm.es (J.L.)
† These authors contributed equally to this work.

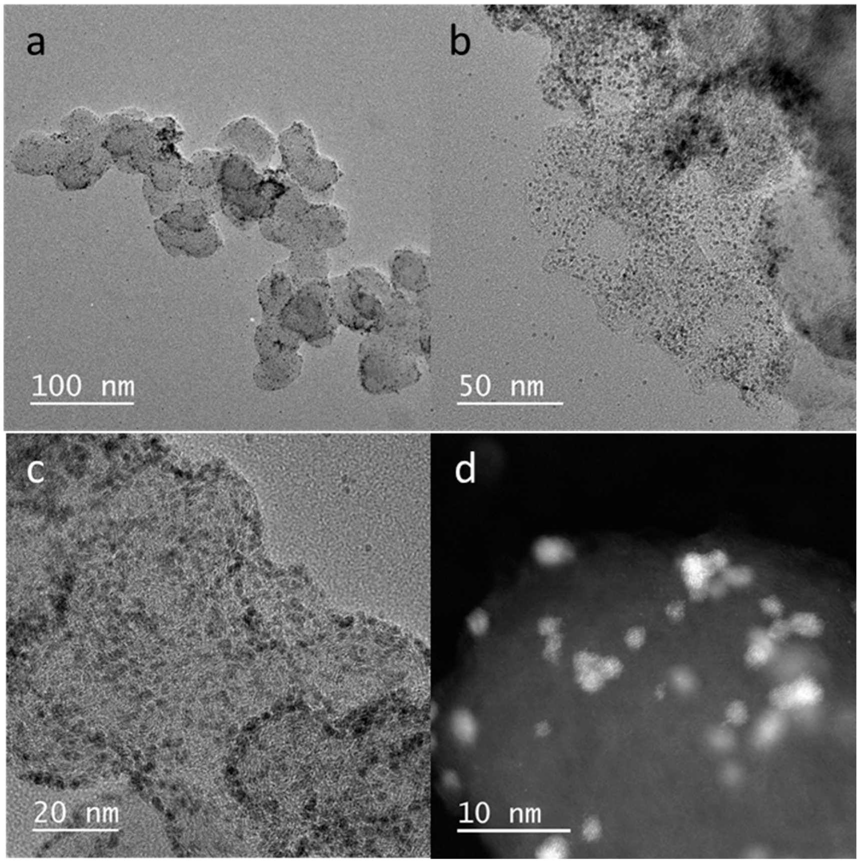

Figure S1. TEM (a,b), HRTEM (c) and STEM-HAADF (d) analysis of RuPt/C-V nanomaterial at different magnifications.

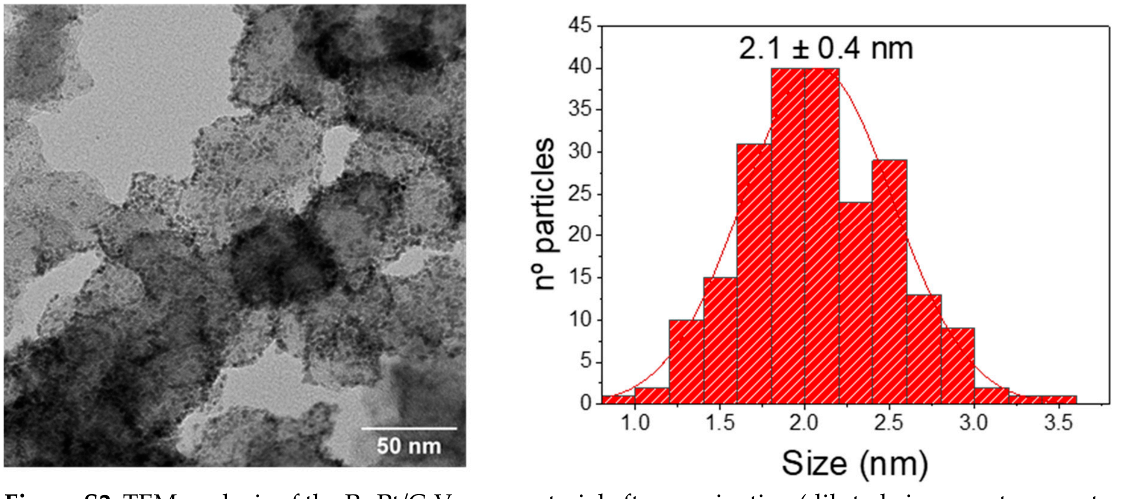

Figure S2. TEM analysis of the RuPt/C-V nanomaterial after passivation (diluted air, room temperature, 3 weeks).

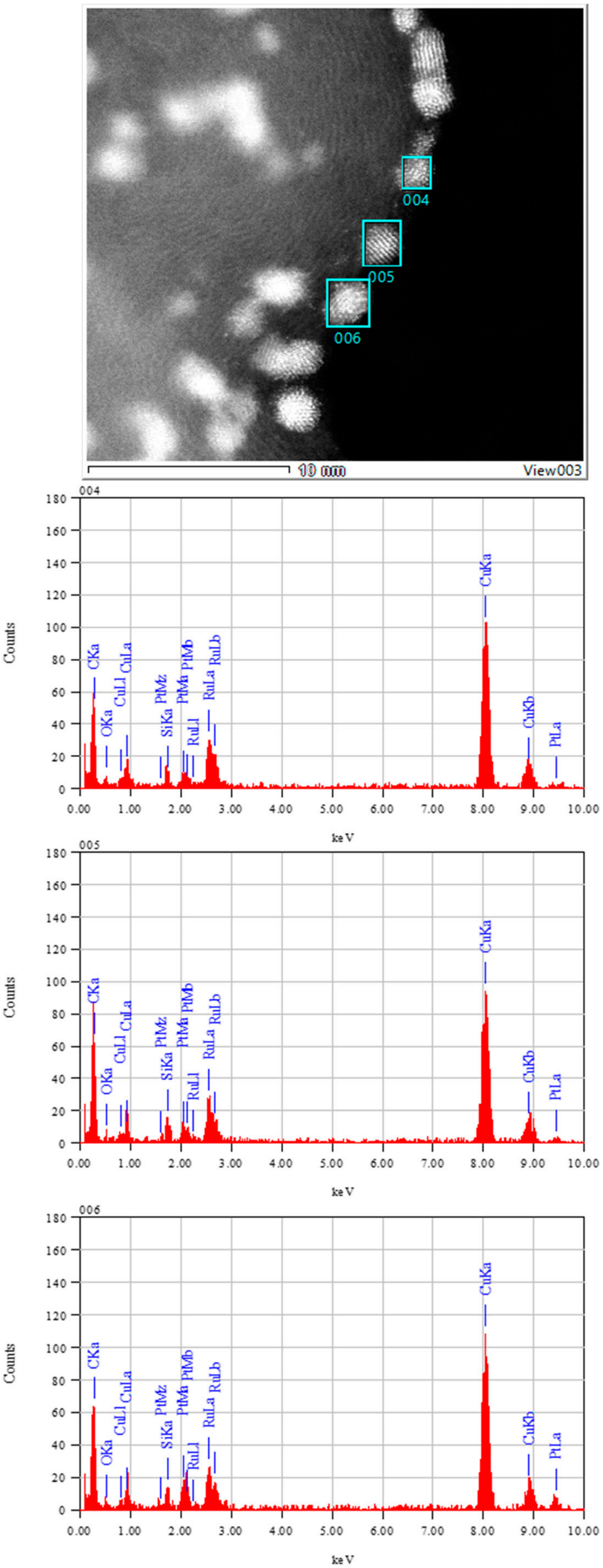

Figure S3. EDX analysis of individual RuPt/C-V nanoparticles depicted on a STEM-HAADF image.

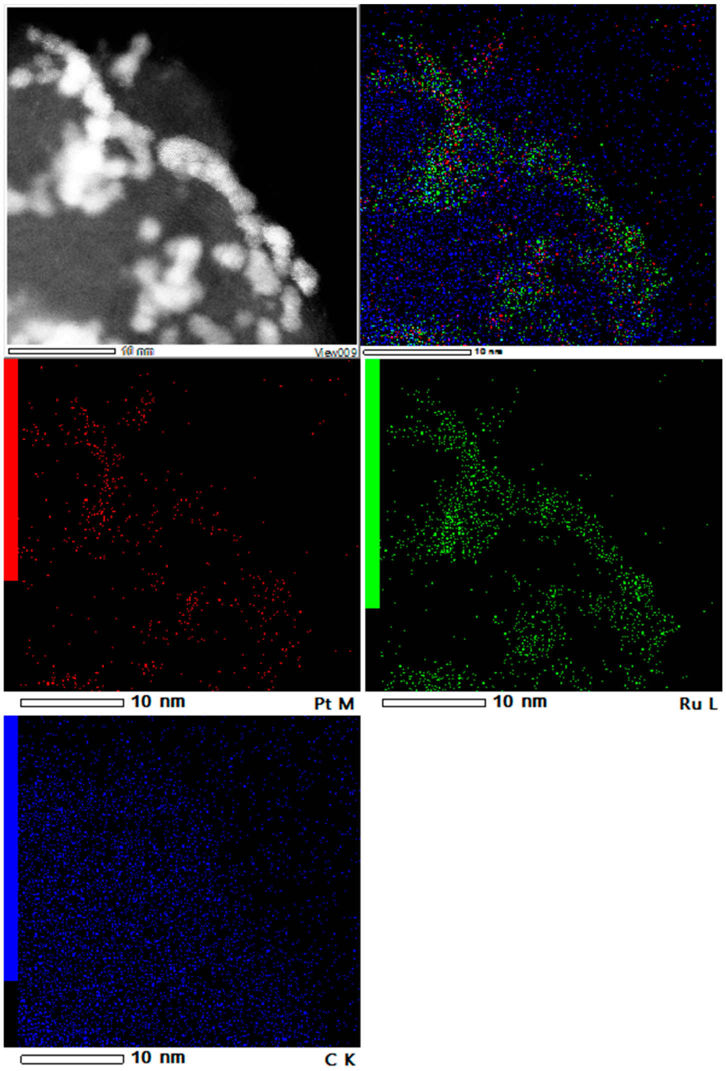

Figure S4. EDX mapping of RuPt/C-V nanomaterial

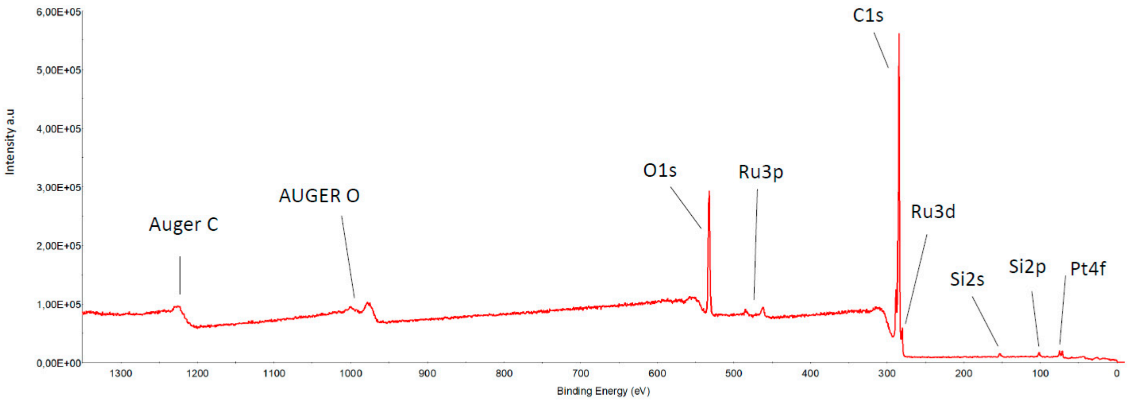

Figure S5. XPS survey of RuPt/C-V nanomaterial .
